# Massive hemothorax due to two bleeding sources with minor injury mechanism: a case report

**DOI:** 10.1186/s13256-018-1813-x

**Published:** 2018-10-07

**Authors:** Koshi Ota, Satoshi Fumimoto, Ryo Iida, Takayuki Kataoka, Kanna Ota, Kohei Taniguchi, Nobuharu Hanaoka, Akira Takasu

**Affiliations:** 1 Department of Emergency Medicine, Osaka Medical College, 2-7 Daigaku-machi, Takatsuki City, Osaka 569-8686 Japan; 2 Department of Thoracic Surgery, Osaka Medical College, 2-7 Daigaku-machi, Takatsuki City, Osaka 569-8686 Japan

**Keywords:** Diaphragm injury, Injury, Hemothorax, Rib fracture

## Abstract

**Background:**

Massive hemothorax resulting from a minor injury mechanism is considered to be rare particularly when the diaphragm is injured. We report a case of massive hemothorax with bleeding from the intercostal artery and diaphragmatic damage caused by minor blunt trauma.

**Case presentation:**

An 83-year-old Japanese man was transported to our hospital 3 hours after falling out of bed. Computed tomography revealed hemothorax and multiple rib fractures. He underwent fluid resuscitation and a tube thoracostomy, but he became hemodynamically unstable. Contrast-enhanced computed tomography revealed worsening hemothorax with contrast extravasation 4 hours after arrival at the hospital. Emergency angiography indicated hemorrhage in the area supplied by the tenth intercostal artery. Transcatheter arterial embolization stabilized his vital signs for a short period. However, further hemodynamic stabilization required a thoracotomy, which revealed diaphragmatic trauma, which was removed and sutured before fixing his fractured ribs. His postoperative course was uneventful, and he was transferred to another hospital for rehabilitation without complications on hospital day 29.

**Conclusions:**

Minor mechanisms of blunt trauma can cause rib fractures and massive hemothorax. Traumatic diaphragm injury should be considered a differential diagnosis if hemodynamic instability persists after transcatheter arterial embolization in patients with lower level rib fractures.

## Background

Major blunt chest trauma often causes massive thoracic bleeds due to multiple rib fractures, as well as pulmonary, cardiovascular, and diaphragmatic injuries. Massive hemothorax resulting from a minor injury mechanism, such as falling from an upright position, is considered to be rare particularly when the diaphragm is injured. To date, only a few case reports have described hemothorax due to phrenic artery injury [1–4]. The present report describes a case of massive hemothorax with bleeding from the intercostal artery and diaphragmatic damage caused by minor blunt trauma, which were treated with transcatheter arterial embolization (TAE) and thoracotomy. The patient provided written, informed consent to publish the details of his condition.

## Case presentation

**Fig. 1:**
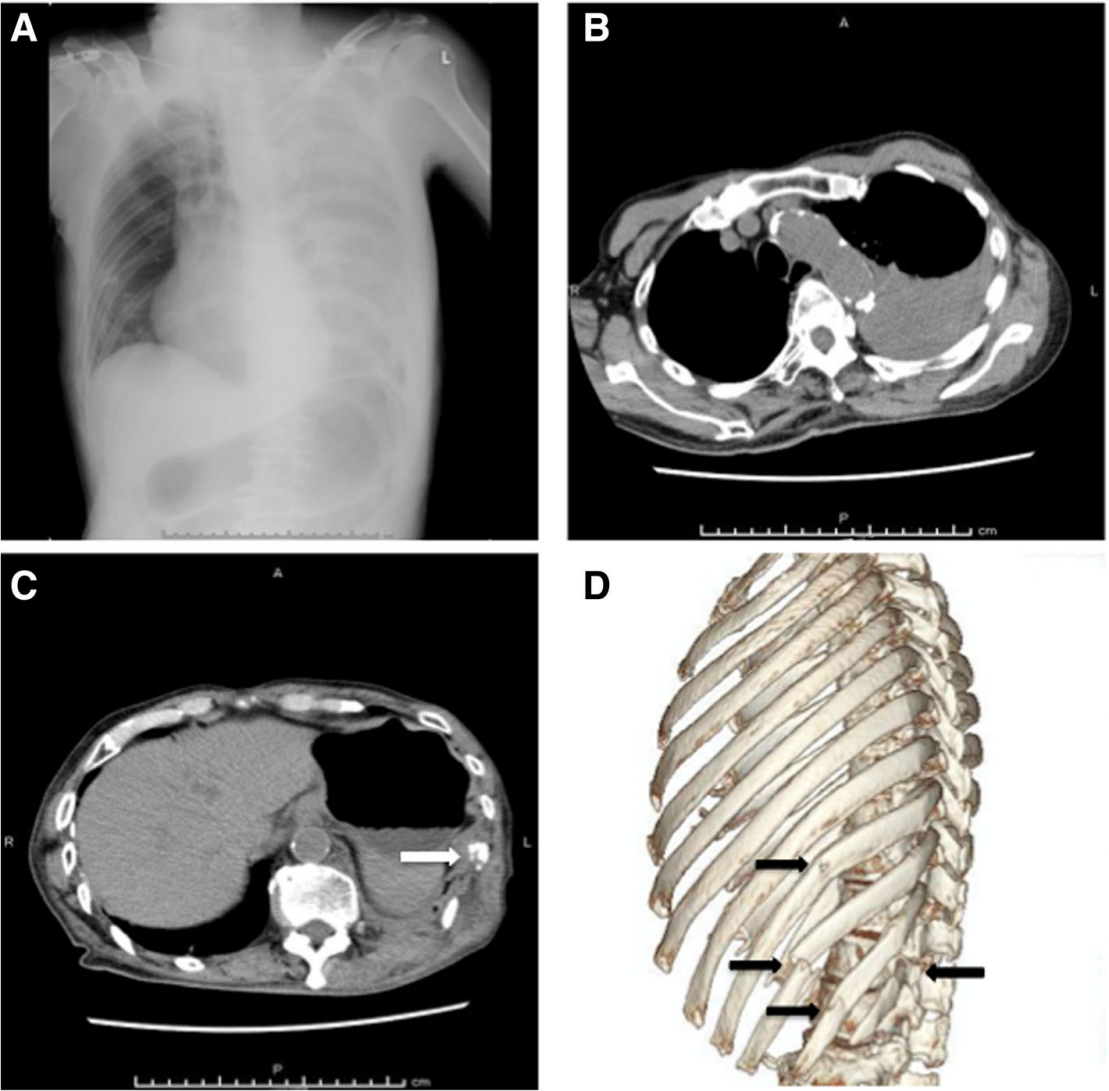
Chest radiography and computed tomography imaging findings of the patient. Chest radiography on arrival at emergency room shows left hemothorax (**a**). First computed tomography images show hemothorax (**b**) and fracture of tenth rib (**c**). Multiple fractures of 9th to 12th ribs (*arrow*) are revealed by (**d**)

**Fig. 2:**
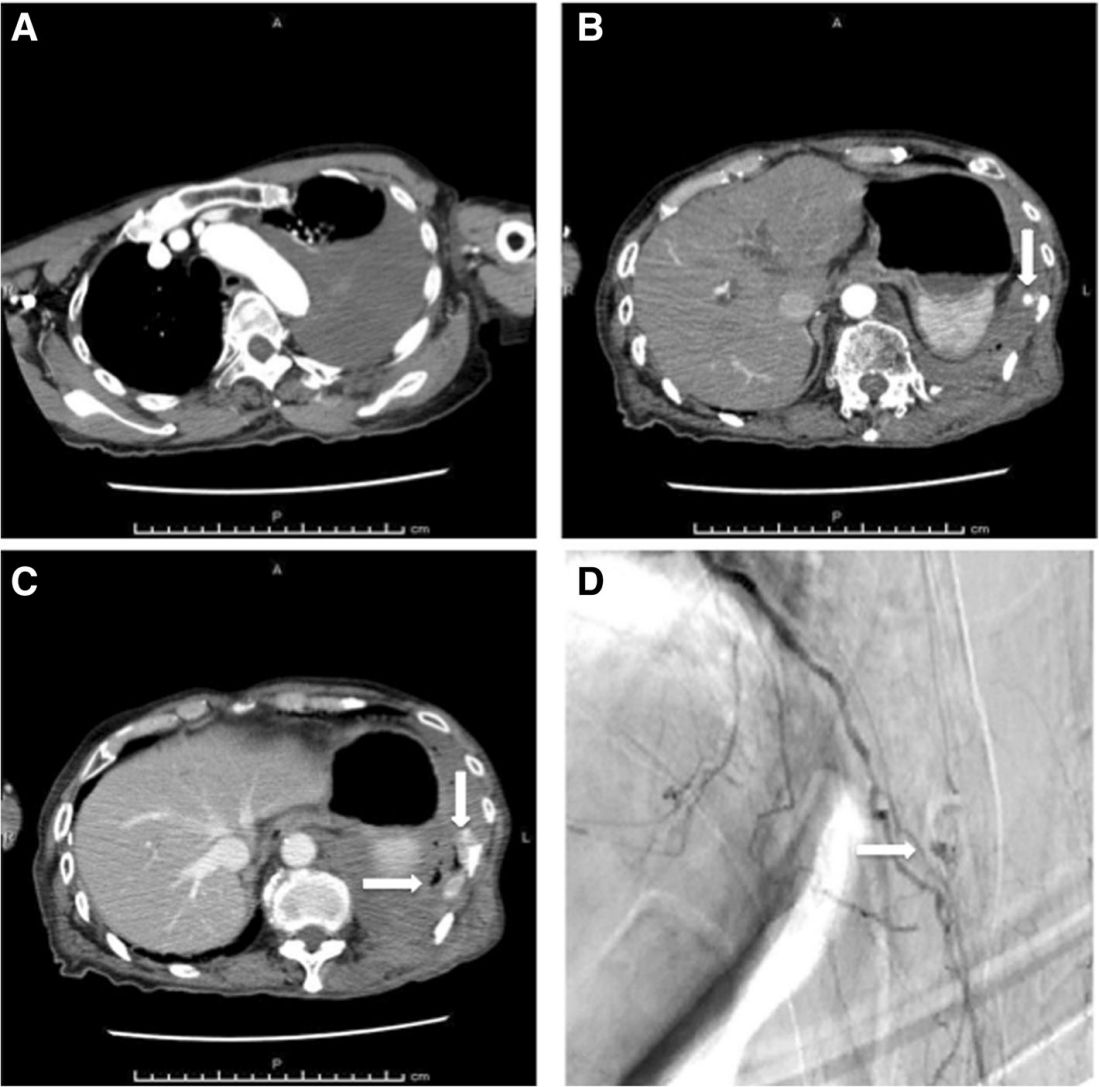
Contrast-enhanced computed tomography and angiography findings. Contrast-enhanced computed tomography (**a**) shows increased hemothorax compared with that shown in Fig. 1b. Early-phase and late-phase contrast-enhanced computed tomography images show extravasation of intercostal artery (*arrow*; **b**) and increased area of extravasation (*arrows*; **c**), respectively, near tenth rib. Angiography (**d**) shows extravasation (*arrow*)

An 83-year-old Japanese man awoke from sleeping and fell out of a bed that was approximately 40 cm high. He called an ambulance 3 hours later because of persistent left-sided chest and back pain and was transferred to our hospital. He had also fallen 3 days before presenting at our hospital and had hit his left arm and the occipital region of his head, for which he had received treatment elsewhere. He had a medical history of cerebral infarction, atrial fibrillation, and prostate cancer, and he had been prescribed apixaban 2.5 mg twice daily and bicalutamide 80 mg/day. His habitual history and familial history were unremarkable. He was a retired medical doctor and lived with his wife’s sister. On arrival at the emergency room, his vital signs were as follows: temperature, 36.2 °C; pulse, 68 beats per minute with an irregular rhythm; respiratory rate, 24 breaths per minute; blood pressure, 143/64 mmHg; and oxygen saturation, 100% on 6 L/minute with a simple oxygen mask. His status on the Glasgow Coma Scale was 13 (E3V4M6), indicating slightly affected consciousness due to mild brain injury. On examination, he was found to be drowsy, pale, and restless. His heart sounds were unremarkable. Cardiac apex was not palpable. His trachea was central and left-sided chest expansion was reduced. There was significant left-sided chest tenderness. Coarse crackles were heard with decreased breath sounds over the left side of his chest. His abdomen was not distended. There was no hepatosplenomegaly. His cranial examination was normal. His limbs examination was normal except for his left arm which had a bruise. Arterial blood gas (ABG) analysis revealed the following: pH, 7.38; partial pressure of carbon dioxide (PCO_2_), 30 mmHg; partial pressure of oxygen (PO_2_), 211 mmHg; bicarbonate (HCO^3−^), 17.5 mmol/L; base excess, − 6.5 mmol/L; hemoglobin, 12.2 g/dL; and lactate, 6.0 mmol/L. Chest radiography and computed tomography (CT) revealed left hemothorax with fractures of the 9th to 12th ribs, which we suspected was associated with the injury sustained during his first fall 3 days before admission (Fig. 1a–d). His blood pressure gradually decreased to 93/45 mmHg after CT assessment, and intensive fluid resuscitation was then initiated. Contrast-enhanced CT (CECT) 4 hours later showed worsening hemothorax with contrast extravasation in the region supplied by the tenth intercostal artery (Fig. 2a–c). A tube thoracostomy at the fifth intercostal space initially drained 950 mL of hemothorax. TAE resulted in hemodynamic stabilization (Fig. 2d). He was admitted to the intensive care unit (ICU) after TAE, but experienced persistent chest tube drainage at a rate of > 200 mL/hour, eventually reaching a total volume of 1820 mL. Measurements of ABG revealed that his hemoglobin had fallen to 7.6 g/dL despite a transfusion with 4 units of red blood cells (RBC).

**Fig. 3:**
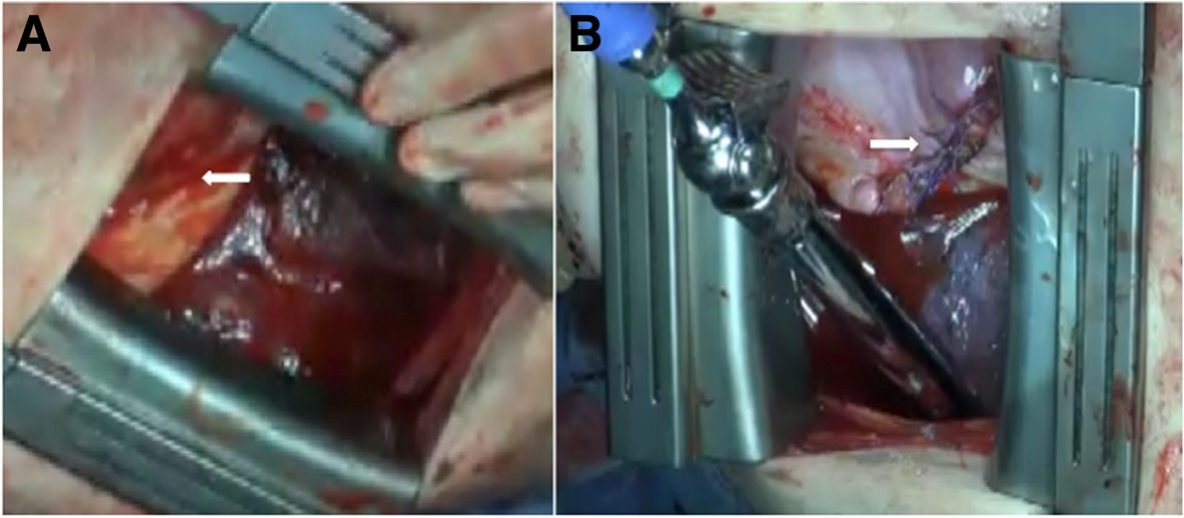
Diaphragmatic trauma. Site of diaphragmatic injury with oozing from phrenic artery branches during surgery (*arrow*; **a**) is removed and site is sutured (*arrow*; **b**)

A thoracotomy at 4 hours after TAE revealed active bleeding from a partial-thickness wound at a peripheral site of his left diaphragm, corresponding to the edge of the broken tenth rib (Fig. 3a). A crushed bleeding lesion was removed from his diaphragm and the site was directly sutured. His fractured ribs were fixed, hemodynamic stabilization was confirmed, and further hemothorax did not arise (Fig. 3b). He required 6 units each of RBC and fresh frozen plasma during surgery. He was discharged from the ICU on hospital day 4 and the chest tube was removed on the following day. His postoperative course was uneventful and he was transferred to another hospital for rehabilitation without complications on day 29. He stayed at a nursing home and received no follow-up visit after discharge. He was managed at the hospital where he used to work and no adverse events were observed at 6 months after discharge.

## Discussion

We described a patient with rare thoracic blunt trauma and two sources of massive bleeding that developed after sustaining a relative minor injury. We considered TAE before thoracotomy because the main source of the bleeding initially seemed to be the intercostal artery. However, thoracic hemorrhage persisted after TAE, and a thoracotomy was required to treat additional bleeding arising from a diaphragm injury. Thoracotomy is quite common and might be required should other bleeding vessels be overlooked at the time of the initial angiography [5]. In our patient, the TAE of the intercostal artery might have partially controlled bleeding due to the diaphragm injury and achieved temporal hemodynamic stabilization because of phrenic–intercostal artery anastomosis [6].

The reported incidence of traumatic diaphragm injury (TDI) ranges from 0.8 to 8% [7]. A diagnosis of TDI is challenging as radiography and CT are insufficiently sensitive [8]. Penetrating TDIs outnumber blunt TDIs by a ratio of approximately 3:1 and mostly result from high-energy impacts caused by motor vehicle collisions [9] rather than relatively low-energy impact caused by minor mechanisms, such as falling a short distance. Patients with blunt trauma sustain injury twice as often on the left side of the body than the right. However, the mortality rate is significantly higher among patients with right diaphragmatic injury than left [7]. Over 50% of patients with TDI have rib fractures, whereas only 0.7% of patients with rib fractures have TDI [9]. A differential diagnosis of TDI should be considered when hemodynamic instability persists in patients with thoracic injuries and fractures of the lower ribs. We initially recognized that the thoracic injury in our patient scored 3 (four rib fractures with hemothorax) on the Abbreviated Injury Scale, but we underestimated its severity because we considered that the hemothorax had been caused by the injury sustained 3 days before admission.

Minor blunt injury, such as a fall from an upright position, can result in serious injuries particularly among elderly persons. Ota *et al.* reported similar findings in a 62-year-old man who had delayed hemothorax due to TDI after a slip and fall [4]. The number of elderly Japanese patients with trauma has remarkably increased with the rapid ageing of the population. One study revealed that falls comprise the most common mechanism of thoracic injury in individuals aged > 70 years and that it results in an increased incidence of fractured ribs and longer hospitalization than that associated with motor vehicle accidents [10]. Elderly patients with rib fractures after a fall might be frailer than other patients, and thus more susceptible to minor mechanisms of injury than those involved in motor vehicle accidents. The possibility of TDI should be assessed when elderly patients present with thoracic blunt trauma accompanied by rib fractures.

## Conclusions

Minor mechanisms of blunt trauma can cause rib fractures and massive hemothorax. Minimally invasive TAE of the intercostal artery is considered the first therapeutic option for elderly patients. However, the intercostal artery is often anastomosed with the phrenic artery as well as other vessels, and rib fractures can result in TDI. TDI should be considered a differential diagnosis if hemodynamic instability persists after TAE in patients with lower level rib fractures.

## Abbreviations

ABG: Arterial blood gas
CECT: Contrast-enhanced CT
CT: Computed tomography
HCO^3−^: Bicarbonate
ICU: Intensive care unit
PCO_2_: Partial pressure of carbon dioxide
PO_2_: Partial pressure of oxygen
RBC: Red blood cells
TAE: Transcatheter arterial embolization
TDI: Traumatic diaphragm injury

## References

[1] Ogawa F, Naito M, Iyoda A, Satoh Y. Report of a rare case: occult hemothorax due to blunt trauma without obvious injury to other organs. J Cardiothorac Surg. 2013;8(1):2–5. 10.1186/1749-8090-8-205. PMC3826551 24176006

[2] Ahn HJ, Lee JW, Kim KD, You IS. Phrenic arterial injury presenting as delayed hemothorax complicating simple rib fracture. J Korean Med Sci. 2016;31(4):641–3. 10.3346/jkms.2016.31.4.641. PMC4810351 27051252

[3] Aoki M, Shibuya K, Kaneko M, *et al.* Massive hemothorax due to inferior phrenic artery injury after blunt trauma. World J Emerg Surg. 2015;10(1):58. 10.1186/s13017-015-0052-3. PMC4657199 26604982

[4] Ota H, Kawai H, Matsuo T. Video-assisted minithoracotomy for blunt diaphragmatic rupture presenting as a delayed hemothorax. Ann Thorac Cardiovasc Surg. 2014;20:911–4. 10.5761/atcs.cr.13-00201. 24200663

[5] Chemelli AP, Thauerer M, Wiedermann F, Strasak A, Klocker J, Chemelli-Steingruber IE. Transcatheter arterial embolization for the management of iatrogenic and blunt traumatic intercostal artery injuries. J Vasc Surg. 2009;49(6):1505–13. 10.1016/j.jvs.2009.02.001. 19497514

[6] Gwon DI, Ko G-Y, Yoon H-K, *et al.* Inferior phrenic artery: anatomy, variations, pathologic conditions, and interventional management. Radiographics. 2007;27(3):687–705. 10.1148/rg.273065036. 17495287

[7] Zarour AM, El-Menyar A, Al-Thani H, Scalea TM, Chiu WC. Presentations and outcomes in patients with traumatic diaphragmatic injury: a 15-year experience. J Trauma Acute Care Surg. 2013;74(6):1392–8; quiz 1611. 10.1097/TA.0b013e31828c318e. 23694863

[8] Fair KA, Gordon NT, Barbosa RR, Rowell SE, Watters JM, Schreiber MA. Traumatic diaphragmatic injury in the American College of Surgeons National Trauma Data Bank: a new examination of a rare diagnosis. Am J Surg. 2015;209(5):864–9. 10.1016/j.amjsurg.2014.12.023. 25952278

[9] Mahamid A, Peleg K, Givon A, Alfici R, Olsha O, Ashkenazi I. Blunt traumatic diaphragmatic injury: a diagnostic enigma with potential surgical pitfalls. Am J Emerg Med. 2017;35(2):214–7. 10.1016/j.ajem.2016.10.046. 27802875

[10] Shi HH, Esquivel M, Staudenmayer KL, Spain DA. Effects of mechanism of injury and patient age on outcomes in geriatric rib fracture patients. Trauma Surg Acute Care Open. 2017;2(1):e000074. 10.1136/tsaco-2016-000074. PMC5887584 29766084

